# A New Phase of the Amalgam Question

**Published:** 1882-10

**Authors:** Melville C. Keith


### SELECTIONS.

### A New Phase of the Amalgam Question.

BY MELVILLE C. KEITH, M.D.

There is a saying to the effect that if one discovers anything of benefit to the race and will not disclose it he is a robber. The writer does not assume to be a discoverer, but many facts coming perforce of necessity under his observation from his profession, have made so profound an impression upon him that it seems justifiable to disseminate the knowledge of these facts for the benefit of humanity. With this preface the writer submits the following:

The *fillings commonly* used by *dentists* for the purpose of *plugging large cavities*, and advocated by a *certain class* of *dentists* to *fill* "*soft, frail teeth*," and *the fillings known under* the *head* of "*Amalgam*," *silver* fillings, and all fillings composed of *cadmium*, *zinc*, *mercury* or *lead*, are *in themselves* directly *injurious* to the *teeth*, to the nerves of the teeth indirectly, and secondarily affecting *injuriously* the *optic and aural nerves*, *causing dimness* of *vision*, deafness, and *softening of the base* of *the brain*.

No one can appreciate the storm of indignant denial which is now being made by the advocates of this "amalgam" or plastic filling, and those interested in the sale of some cement, and also from those who will reiterate the often repeated assertion that "tons of amalgam are being carried around in people's heads," and no one makes complaint. In order to ascertain whether there is any complaint from "amalgam" fillings, it is only necessary to examine the very large and increasing number of those who have neuralgia, weak eyes, and are *partially deaf* from the effect of amalgam filling. These cases are referred to the oculists, the aurists, or the physicians, many of whom are too utterly careless of the benefits derived from proper dental hygiene. To-day many hundreds of agonizing neuralgias, weak eyes, and ringing in the ears, would be radically cured by an application to a scientific dentist, rather than to the physician who almost universally resorts to opium in one form or another to lull the pain instead of *removing the cause*.

The injurious effect of mercury and zinc, as fillings of teeth, arises from the fact that these metals, *with the saliva*, form a *galvanic current* in the mouth, immediately affecting the nerves of the pulp chamber, thence the dental and maxillary nerves, and, by immediate relation, affecting the trifacial (fifth pair), and thence *all* the cranial nerves.

Now, in relation to two metals, *with the acid of the saliva* forming a current or a galvanic battery. The first query of the unscientific mind would be, "How can this prove to be a galvanic current and yet no shock be felt?" The correct reply would be, "because, first, the metals are in contact; second, because these metals, with an acid *always* form a current, and it would not make any difference whether the conditions were on the top-most peak of the Rocky Mountains or in Africa, or in a man's mouth or teeth. *Like causes produce like results*, and the conditions of the current being present, the current is certainly present.

In explanation of the question why no shock is felt, the reply is because both currents are connected and present. For instance, take the galvano-faradio No. 3, or No. 4 electro-magnetic battery, and with a freshly charged cell have all the power (electricity) possible. If, now, both poles or sponges are placed in one hand, and the sponges *touch one another*, no current can be felt. But take each sponge in different hands and an ordinary person cannot bear the shock. So with the amalgam fillings of the teeth; both currents are together and cannot be appreciated at the point of contact. If, however, two different dentists have placed two different kinds of amalgams or metals, the current becomes appreciable to certain sensitive people to such an extent that one filling has to come out.

Many a milliner or dressmaker, in the habit of placing pins or needles in their mouths, can testify to the sudden dart of exquisite pain arising from the contact of brass or steel with the mercurial and zinc filling. Nor is it necessary to have the current felt or broken to be effective.

Reynolds says (page 20, Clinical Uses of Electricity): "The chemical action of faradization is almost *nil*; the direct effect on temperature is almost *nil*; it causes no burning feeling, like that which is communicated by the galvanic current, but under ordinary circumstances it produces marked contraction of the muscles."

Althaus (Medical Electricity, page 130, 3d edition): "The only form of electricity which, if applied in moderate intensity, has a distinct physiological action on the brain, of the living man, is the continuous current. Static electricity, electro-magnetism, and magneto-electricity only affect that organ if applied so powerfully as to interfere with health and perhaps life; but a gentle, continuous current, directed to the face, scalp or neck, and which causes no, or scarcely any, sensation of pain, is readily transmitted from those parts to the cerebral substance."

From these quotations it appears that faradization, induced current, and a continuous current can be present and effective and yet quite imperceptible. Furthermore, after the teeth have been filled with amalgam, the teeth so filled turn black from the *chemical action* of the zinc and mercury, which is, in itself, electricity. "Wherever you have chemical decomposition in progress, there is also some electrical change going on." Reynolds, page 15.

The question cannot be answered by personal invectives against scientists. It is a question which is of paramount importance to the dental profession, and one that must be answered in the affirmative each time the question is asked, "Do amalgam fillings form with the acid of the saliva a battery or a galvanic current in the mouth?" Further examination corroborates this assertion. The usual formula for the ordinary battery is zinc, platinum, and dilute sulphuric acid. The Storhrer battery (or what is better known in America, the Galvano-Faradio Manufacturing Company's batteries), is a current induced from carbon and zinc in a solution of sulphuric acid and bi-chromate of potash. In these batteries the zinc is soon decomposed. If the amalgam fillings for the teeth were composed of pure zinc, and the saliva dilute sulphuric acid, we could demonstrate the most powerful currents by showing the rapid decomposition of the zinc. As a matter of fact, the zinc is in the tooth, the acid (in the saliva) is present, and a *current must exist.* The reason why the zinc is not decomposed is because the zinc is most *thoroughly amalgamated* with quicksilver by the dentist before placing it in the victim's tooth. For a corresponding action, let one observe the Jerome Kidder batteries, say a No. 5. The formula for the current generated by this most elegant galvanic battery is carbon, platinum, one part sulphuric acid, twelve parts water. The directions are: "Put a tablespoonful of mercury in the bottom of the jar." Though the most powerful current can be generated by this battery, the zincs, while they touch the mercury, look fresh and bright. If, however, the mercury is not put in, the zinc turns a blackish brown dirty color; *and the same condition is seen in many fillings* of *teeth,* varying according to the amount of mercury placed with the zinc at the time of filling the teeth.

If we follow the eminent American authorities, Messrs. Beard and Rockwell, this current would come under the head of "Dynamical Electricity," arising from the dissolution of metals, for "it is found that chemical electricity is most conveniently generated by the reaction that takes place between two metals and some acid solution, and as a matter of economy, zinc is the metal at the expense of which the electrical force is evolved, the other metals acting merely as conductors. (See Medical Electricity, page 19.)

It is, however, the province of physicist and electricians to demonstrate whether the current, from a zinc and mercury filling is "induced," "faradic," or "dynamical," or whether the action is "voltaic," "electro-magnetic," or "galvanic," it is our place to decide that any and all excessively continuous currents are dynamically injurious to all the cranial system of nerves. How the current from an amalgam filling is generated and carried to different points, how it causes headache, dimness of sight, depression of mental power, is safely left to be discussed in the future. We have said enought to show that every amalgam filling, *per se,* with an acid, and the acids certainly are not wanting in these days of acetic acid, and sulphuric acid vinegars, or in the sugars from glucose and sulphuric acids so commonly retailed, or indeed from all Americanized syrups manufactured from corn or sorghum. The most eminent electricians of America, Beard and Rockwell (*op. cit.*, page 26) says: "Just how the current is formed we do not fully know; we know that when the different metals touch each other, the positive electricity will go to one metal and the negative to another." Writing of the Leyden jar these authors say: "*When the metals are made to touch each other or are connected with wires*, they are relieved of their charge and again become charged, then again relieve themselves, and so on indefinitely. There is no equilibrium established, but a constant effort to establish it which never succeeds. This constant effort to establish an equilibrium keeps up the current."

Any one conversant with the ordinary facts of chemistry can decide whether with zinc, mercury, and an acid saliva, there is or is not a current in the tooth or mouth affecting the entire buccal cavity. To the unfortunate victims who have amalgam fillings, this current of electricity is a daily demonstrated fact. The *taste* the first few days after the tooth was filled, was from a *current of electricity*. The *inflammatory condition of the gum* was from a current of electricity *generated* by the action of the zinc, mercury, and an acid saliva. It will not answer the purpose to declare that "frail soft teeth" can be saved with amalgam. If that amalgam constitutes an electrical current, which is destructive to the eyes, the ears, and to the base of the brain, from a current being transmitted to the origin of the fifth pair, and if, as has been shown, the amalgam fillings constitute a force sufficiently powerful to destroy the intellectual attributes of the man or woman who is unfortunate enough to be victimized by an ignorant or unthinking dentist, then it becomes a matter for the people themselves to investigate, and enact such laws as may prevent the ignorant ones from having this nerve destroying compound placed in their mouths, even though that destructive process is under the guise of saving "frail soft teeth," or more ostensibly to benefit the sellers of amalgam, and the authors of plastic filling. Those who have not investigated this subject, we will refer to the work already cited, "Medical and Surgical Electricity" As to the definition of electricity, which is there defined, to be "a *disturbance* propagated in the molecules of a body and the ether pervading that body," (page 27) and this "disturbance" from zinc and mercury, with an acid saliva, is a disturbance which intelligent people will no longer submit to. We will now examine the *effect* of this "disturbance" on the maxilliary nerves.

---

MEDICAL men are very plentiful in the United States compared with other countries: 1 to every 600 inhabitants, while Canada has only one to 1,200 inhabitants; Great Britain 1 to 1,672; Germany 1 to 3,000. A higher standard of examination is recommended, and an all-round education insisted upon. Only five schools at present require the highest amount of study to qualify a full practioner.

